# The Science and Art of Obstetrics

**Published:** 1887-03

**Authors:** 


### Reviews.

**The Science and Art of Obstetrics.** By THEOPHILUS PARVIN, M. D., LL. D., Professor of Obstetrics, etc., in Jefferson Medical College. Illustrated with two hundred and fourteen wood cuts and a colored plate. Philadelphia: Lea Brothers & Co. 1886.

The author states in the preface that he has endeavored to write a book which will be useful alike to students and to practitioners. In carefully examining this work, we conclude that he has been eminently successful in preparing a guide in obstetric medicine to students, and we doubt not that like success has attended his efforts for the practitioner. In our experience with medical classes, the need of a text-book which would be both comprehensive and yet within a moderate compass has been felt. The present work includes the result of a large experience in obstetrics, which is presented as only a teacher knows how to unfold the subject to minds eager to grasp general principles; also, to remember details as far as memory would permit. Prof. Parvin has succeeded in this difficult task better than any other author with whom we are familiar, and the present work is admirably adapted for the two classes for which it was prepared. It also contains the latest advances in this science, while showing a wise conservatism in holding fast to principles established by past experience.

The work is too complete to require an analytical review. The high professional standing of the accomplished author, and his success as a teacher, give assurance that he has bestowed upon its preparation the more careful labor as well as the result of a large experience.

It may be said that while the profession possess such works as Barnes and Playfair and Lusk on this subject, further efforts are superfluous; we object to this view. We find in the various works features which show the individuality of the author. The present work is singularly free from "hobbies," while presenting the subject in a clear and concise manner, which will make it valuable as a text-book in medical classes.

In looking over the pages, we think Prof. Parvin has performed a valuable service for the profession and we commend his labor.

